# The transmission of drug-resistant strains of HIV in heterosexual populations based on genetic sequences

**DOI:** 10.1371/journal.pone.0259023

**Published:** 2021-12-01

**Authors:** Xin Jin, Zhen Wang, Zhiyuan Zhang, Hui Wu, Yuhua Ruan, Chen Zhang, Ruihua Kang, Hui Xing, Jie Lou

**Affiliations:** 1 Department of Mathematics, Shanghai University, Shanghai, China; 2 Department of Statistics, Columbia University, New York, NY, United States of America; 3 State Key Laboratory for Infectious Disease Prevention and Control, Collaborative Innovation Center for Diagnosis and Treatment of Infectious Diseases and National Center for AIDS/STD Control and Prevention, Chinese Center for Disease Control and Prevention, Beijing, China; 4 School of Nursing, University of Rochester, Rochester, NY, United States of America; 5 Department of Disease Prevention and Control, The Affiliated Cancer Hospital of Zhengzhou University, Henan Cancer Hospital, Zhengzhou, China; University of Cincinnati College of Medicine, UNITED STATES

## Abstract

**Background:**

China’s National Free Antiretroviral Treatment Program (NFATP) has substantially reduced morbidity and HIV/AIDS incidence since 2003. However, HIV resistance to antiretroviral drugs (ARVs) has been a major challenge for the current treatment of HIV/AIDS in China.

**Methods:**

In the current study, we established a nested dynamic model to predict the multi-drug resistance dynamics of HIV among the heterosexual population and evaluated the impact of intervention measures on the transmission of drug resistance. We obtained an effective reproductive number $R_{e}^{d}$ from each sub-model held at different stages of the dynamic model. Meanwhile, we applied Bayesian phylogenetic methods to infer the weighted average effective reproductive number $R_{e}^{g}$ from four HIV subtypes that sampled from 912 HIV-positive patients in China. It is an original and innovative method by fitting $R_{e}^{d}$ to $R_{e}^{g}$ by Markov Chain Monte Carlo (MCMC) to generate unknown parameters in $R_{e}^{d}$.

**Results:**

By analyzing the HIV gene sequences, we inferred that the most recent common ancestor of CRF01AE, CRF07BC, CRF08BC, and CRFBC dated from 1994, 1990, 1993 and 1990, respectively. The weighted average effective reproductive number $R_{e}^{g}$ dropped from 1.95 in 1994 to 1.73 in 2018. Considering different interventions, we used a macro dynamic model to predict the trend of HIV resistance. The results show that the number of new infections and total drug resistance under the baseline parameter (**S**_**1**_) are 253,422 and 213,250 in 2025, respectively. Comparing with the numbers under the target treatment rate (**S**_**2**_), they were 219,717 and 236,890, respectively. However, under the ideal treatment target (**S**_**3**_, the treatment rate reaches 90% and the treatment success rate reaches 90%), the number of new infections shows a declining trend and will decrease to 46,559 by 2025. Compared with **S**_**1**_ and **S**_**2**_, the total number of resistance also decreased to 160,899 in 2025.

**Conclusion:**

With the promotion of NFATP in China, HIV resistance to ARVs is inevitable. The strategy of increasing the treatment rate would not only ineffectively curb the epidemic, but also deteriorate drug resistance issue. Whereas, a combination of intervention strategies (the treatment rate reaches 90% and the treatment success rate reaches 90%) can greatly reduce both infection and drug resistance rate than applying one strategy alone.

## Introduction

Acquired Immune Deficiency Syndrome (AIDS) is one of the biggest threats to public health in the world. Although the Chinese government has made great efforts in preventing and controlling the epidemic, the current situation is concerning [1]. Currently, heterosexual transmission is the main route of HIV/AIDS transmission in China. According to the most recent report from the China CDC, heterosexual transmission accounted for 73.7% of the new HIV/AIDS infections from January to October 2019 [2].

To effectively curb the HIV/AIDS epidemic in China, the Chinese government has implemented “Four Frees and One Care” policy since 2003 [3, 4]: provide free highly active antiretroviral therapy (HAART) for HIV infected individuals who are eligible for treatment. The promotion of HAART has significantly reduced morbidity, mortality, transmission and new infections of HIV/AIDS [5, 6]. However, increasing the treatment rate also led to an increase in the spread of various HIV-resistant strains, which is the main reason for the failure of antiretroviral therapy (ART) and becomes a major challenge in HIV/AIDS treatment [7]. Growing evidence shows that HIV drug resistance is rising rapidly in China, which greatly hindered the efforts made by the government and health professionals [8–10]. Therefore, understanding the transmission of HIV resistance among heterosexuals can provide important direction for future ART programs.

On the other hand, the research on HIV resistance in China is mainly focused on a certain region (province or city), and the research is conducted on the traditional monitoring data from a single province, city, or a single medical institution [11–17]. In this paper, we combined microscopic HIV molecular sequence information with the macroscopic epidemiological dynamic system to analyze the drug-resistant transmission of HIV. Based on the Bayesian inference framework and Markov Chain Monte Carlo (MCMC) sampling method, our work is to study the transmission dynamics of HIV resistance and predict the trend of HIV resistance for the next few years.

## Materials and methods

### Ethics statement

The ethics approval and consent to participate was granted by the National Center for AIDS/STD Control and Prevention (NCAIDS), Chinese Center for Disease Control and Prevention (China CDC).

### Data

The genetic data studied in this paper was sampled from HIV-positive patients who were infected via heterosexual transmission and diagnosed in the past 1–2 years in various provinces across the country. The genetic data was sampled between July 2016 and October 2017. The drug resistance was analyzed by the algorithm of the Stanford HIV Drug Resistance Database (HIVDB, Version 8.4) (https://hivdb.stanford.edu/hivdb/by-sequences/). And the definition of any drug resistance is defined with respect to one or more the following drugs or drug classes: EFV, NVP, any NRTI, DRV/r, LPV/r or ATV/r. Classifications of “susceptible or potential low-level” are considered as no drug resistance (a Stanford penalty score <15) and at least low-level is defined as drug resistance (a Stanford penalty score ≥ 15). Through data cleaning, we obtained 912 HIV-pol gene sequences in total. We selected CRF01AE (350), CRF07BC (322), CRF08BC (206), CRFBC (34) as the four subtypes for further study since they have sufficient sampling size. CRFBC is a recombinant strain of subtypes B and C. More details given on data cleaning, subtype classification, or parameters of the multiple sequence alignment can be found in the literature (including study design and study participants, data collection and laboratory tests) [18]. The gene sequence dataset of these four subtypes was aligned by using Mega software [19]. Particularly, the total length of the studied gene sequence was 1038bp.

### Bayesian phylogenetic analysis

In this paper, we applied Bayesian evolutionary analysis by sampling trees (BEAST2) to perform Bayesian phylogenetic analysis on HIV sampling sequences [20, 21]. The core algorithm of this method is the Metropolis-Hasting Markov Chain Monte Carlo sampling algorithm [22, 23]. In this study, we selected birth-death skyline plot (BDSKY) to analyze the origin of gene sequence data and related epidemiological parameters [24]. The Birth-death skyline diagram is a forward time model based on transmission, death/recovery and sampling, and these parameters are allowed to change in a piecewise constant fashion. More importantly, the model quantifies an important epidemiological parameter: the effective reproductive number $R_{e}^{g}$ [25]. $R_{e}^{g}$ reflects the dynamic process of virus transmission. Thus, we can use the transmission parameter $R_{e}^{g}$ inferred from the genetic data to fit the unknown parameters in dynamic models.

During the phylogenetic analysis, the selection of an evolutionary model (also called as nucleotide substitution model) is a critical step. In the current study, we used Phylosuite to select the nucleotide substitution model of the sequences data to be analyzed [26]. The model selection process is based on Akaike Information Criterion (AIC) (a model with a lower AIC score is more suitable) [27]. After calculating the AIC score, *HKY* + Γ + *I* model was chosen to be the nucleotide substitution models of the four subtype sequences, and we set a strict molecular clock. The clock rates of the four subtypes are set to 0.005, 0.00255, 0.003 and 0.00255, respectively. See Table 1 in the S1 File for prior information setting of the BDSKY model parameters.

### Dynamic model

Considering the heterosexual transmission of HIV virus with drug resistance, we divided the resistance into single-drug resistance, dual-drug resistance and triple-drug resistance. Furthermore, the compartments were divided into three categories: susceptible individuals, untreated infected individuals and treated infected individuals (the infected individuals include drug-sensitive and drug-resistant individuals). Given the fact that HIV infection is a long and complex process, and taking account of the national treatment standards for implementation of the free HIV treatment policy, we divided the infection process into three stages according to CD4 T cell count in patients: CD4 count > 500 cells/*μl* (Stage 1), 200 cells/*μl* ≤ CD4 count ≤500 cells/*μl* (Stage 2), CD4 count<200 cells/*μl* (Stage 3, the AIDS phase).

There are three main types of first-line antiretroviral drugs in China [28]: Nucleoside Reverse Transcriptase Inhibitor (NRTI) [29], Non-Nucleoside Reverse Transcriptase Inhibitor (NNRTI) [30] and Protease Inhibitor (PI) [31–33]. In this paper, we only considered the resistance of these three main drugs in the model. Suppose that resistance is divided into single-drug resistance (only resistance to one type of drug in NRTI, NNRTI or PI), dual-drug resistance (resistance to any two types of drugs in NRTI, NNRTI and PI) and triple-drug resistance (resistance to NRTI, NNRTI and PI at the same time), the infected individuals can be divided into four categories according to the drug resistance: non-drug resistance(or drug sensitivity), single-drug resistance, dual-drug resistance and triple-drug resistance. We also consider primary resistance and secondary resistance. Primary resistance can also be called as transmission resistance because such drug-resistant strains also have a certain transmission capacity, and susceptible individuals can be directly infected with resistant strains. However, secondary resistance is usually caused by non-transmission factors such as selective pressure of drugs, mutated viruses, poor drug compliance, drug interactions, and pharmacokinetics caused by infected patients after receiving antiretroviral therapy [34]. We assumed that the patients first developed resistance to a single drug with a certain probability, then to a dual-drug resistance, finally to a triple-drug resistance. And for patients with single or dual resistance, we consider changing their treatment plan for further treatment, but treatment is no longer considered for triple resistance cases.

The government of China introduced the “four frees and one care” HIV free treatment policy in 2003. In the other words, there was no government support for HIV-free treatment before 2003 (recorded as the period I). Since the implementation of this policy, there have been three important changes in the national standard of free antiviral treatment: from 2003 to 2011 (recorded as period II), antiviral therapy was provided when the CD4 cell count < 200/*μl* in the patient [35, 36]; from 2012 to 2015 (recorded as period III), antiviral treatment was provided when the CD4 cell count <500 /*μl*, and from 2016 to now (recorded as period IV), the treatment is performed once the patient is diagnosed.

**Fig 1:**
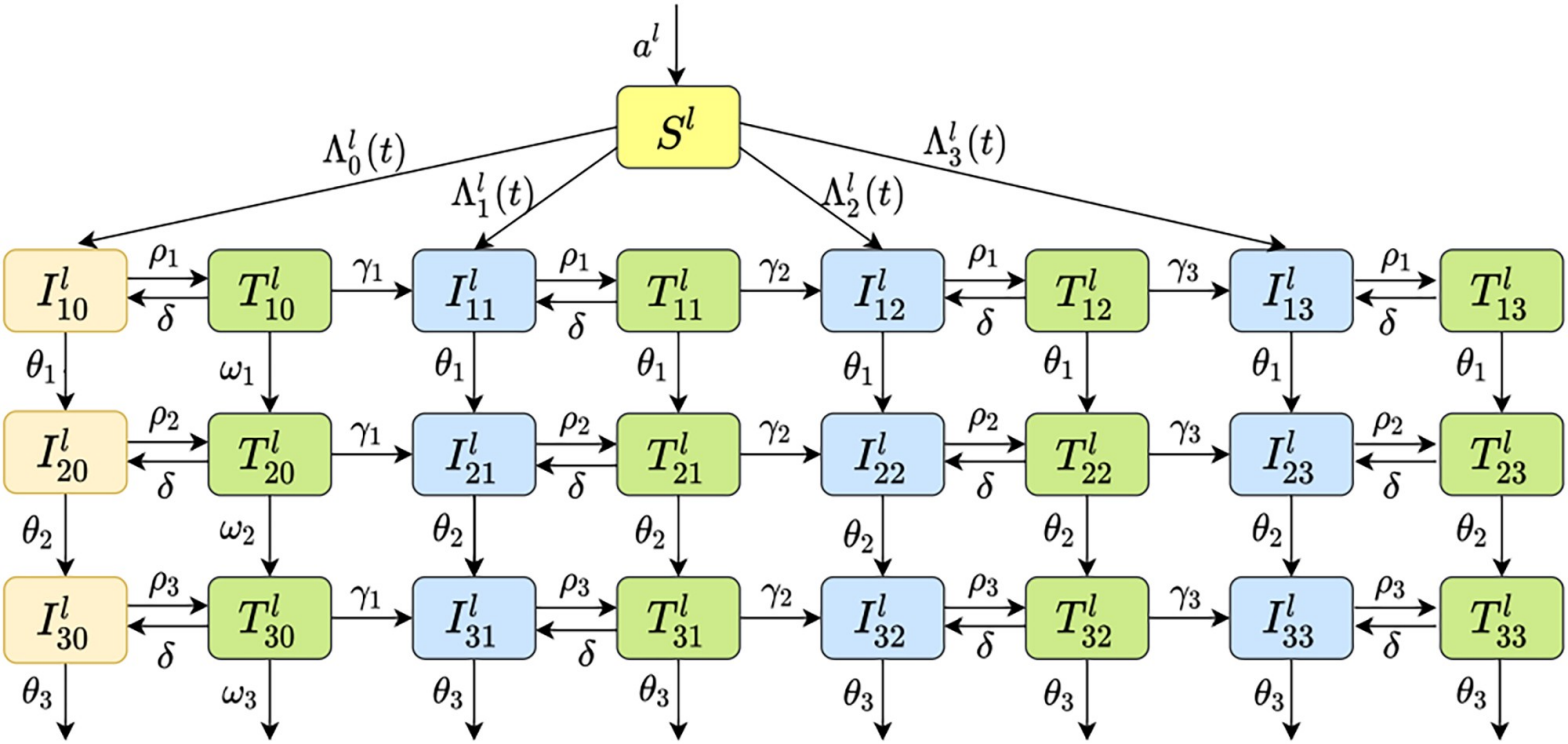
HIV transmission diagram.

Based on these assumptions, we established a nested dynamic model for HIV transmission and resistance in heterosexual population, which includes four different models from simple to complex based on four different history periods mentioned above. We used *S* for susceptible individuals, *I* for untreated infected individuals, and *T* for infected individuals under treatment. There are two subscripts for the two infected categories: *I*_*ij*_ and *T*_*ij*_. The subscript *i* ∈ {1, 2, 3} is used to represent three different infection stages, and subscript *j* ∈ {0, 1, 2, 3} is used to indicate the type of drug resistance, representing no drug resistance (*j* = 0), single-drug resistance (*j* = 1), dual-drug resistance (*j* = 2) and triple-drug resistance (*j* = 3), respectively. Superscript *l* is used to indicate gender, where *l* = *w* represents women, and *l* = *m* represents male. The compartment diagram of the model is shown in Fig 1. The compartment diagram is based on the period IV (the treatment was performed once the patient is diagnosed). The corresponding dynamic equations are shown in the Eq 1. For the four different historical treatment period I, II, III and IV, we can simplify Fig 1 based on its history characters. The corresponding assumptions of the four sub-models can be found in Table 1.

**Table 1:** Sub-models and corresponding assumptions.

Symbols of sub-models	Assumptions	The corresponding historical periods
I	*ρ*_*i*_ = 0, *δ* = 0, *γ*_*i*_ = 0, *ω*_*i*_ = 0	1994–2002
II	*ρ*_1_ = 0, *ρ*_2_ = 0, *ω*_1_ = 0, *ω*_2_ = 0	2003–2011
III	*ρ*_1_ = 0, *ω*_1_ = 0	2012–2015
IV	Full model	After 2016

Note. i = 1,2,3, which represents infection stage in the full model.

In this system, *a* is an input of susceptible individuals, *θ*_*i*_, *i* = 1, 2, 3 are the disease progression rates of sensitive strains carriers without receiving treatment and resistant strains carriers receiving treatment in infection stage 1, 2 and 3, respectively. *ω*_*i*_, *i* = 1, 2, 3 are the disease progression rates of sensitive strains carriers receiving treatment. *ρ*_*i*_, *i* = 1, 2, 3 are the treatment rates of patients in stage 1, 2 and 3. *γ*_*i*_, *i* = 1, 2, 3 are the single-drug resistance rate, dual-drug resistance rate and triple-drug resistance rate caused by drug treatment. Finally, *δ* is the withdrawal rate from treatment.

$$\begin{array}{c} \left\{ \begin{array}{cc} & \frac{dS^{l}}{dt}=a^{l}-\sum_{j=0}^{3}\Lambda_{j}^{l}\left(t\right)-\mu S^{l},\\ & \frac{dI_{ij}^{l}}{dt}=\Lambda_{j}^{l}\left(t\right)+\gamma_{j}T_{i,j-1}^{l}+\delta T_{ij}^{l}-\left(\rho_{i}+\theta_{i}+\mu \right)I_{ij}^{l},\;\;\;\;\;\;i=1;j=0,1,2,3\\ & \frac{dI_{ij}^{l}}{dt}=\theta_{i-1}I_{i-1,j}^{l}+\gamma_{j}T_{i,j-1}^{l}+\delta T_{ij}^{l}-\left(\rho_{i}+\theta_{i}+\mu \right)I_{ij}^{l},\;\;\;\;i=2,3;j=0,1,2,3\\ & \frac{dT_{ij}^{l}}{dt}=\omega_{i-1}T_{i-1,j}^{l}+\rho_{i}I_{ij}^{l}-\left(\delta +\gamma_{j+1}+\omega_{i}+\mu \right)T_{ij}^{l},\;\;\;\;\;\;\;\;i=1,2,3;j=0\\ & \frac{dT_{ij}^{l}}{dt}=\theta_{i-1}T_{i-1,j}^{l}+\rho_{i}I_{ij}^{l}-\left(\delta +\gamma_{j+1}+\theta_{i}+\mu \right)T_{ij}^{l},\;\;\;\;\;i=1,2,3;j=1,2,3 \end{array}\right. \end{array}$$ (1)

In the above equation, *γ*_0_ = *γ*_4_ = *ω*_0_ = *θ*_0_ = 0. $\Lambda_{j}^{l}\left(t\right),\left(l\in \left\{m,w\right\},j\in \left\{0,1,2,3\right\}\right)$ represents the number of newly infected individuals in the group *l* infected with the *j* type of HIV strain at time *t*. And we denote *N*^*l*^ for the total population of the group *l*. The details are as follows:

$$\begin{array}{c} \begin{array}{cc} & \Lambda_{j}^{m}\left(t\right)=\frac{c^{m}S^{m}\sum_{i=1}^{3}k\lambda_{j}\beta_{ij}\left(I_{ij}^{w}+\epsilon_{i}T_{ij}^{w}\right)}{N^{w}},\;j=0,1,2,3\\ & \Lambda_{j}^{w}\left(t\right)=\frac{c^{w}S^{w}\sum_{i=1}^{3}\lambda_{j}\beta_{ij}\left(I_{ij}^{m}+\epsilon_{i}T_{ij}^{m}\right)}{N^{m}},\;j=0,1,2,3\\ & N^{l}=S^{l}+\sum_{i=1}^{3}\sum_{j=0}^{3}\left(I_{ij}^{l}+T_{ij}^{l}\right),\;i=1,2,3;\;j=0,1,2,3 \end{array} \end{array}$$

Here, *c* represents average number of partners per year. *k* is infection rate ratio coefficient of female with respect to male. λ_*j*_ is used to explain proportional coefficient of infection rate of *j* resistant strain carriers. *β*_*ij*_ represents infection rate of *j* strains carriers in the *i* stage of infection. *ϵ*_*i*_ is the probability of transmission was reduced after the *i* infection stage treatment.

## Results

### Results of Bayesian inference—Retrospect the transmission dynamics of HIV Virus

We analyzed the sequence from four different HIV-1 subtypes with clear collecting time sampled from 912 HIV-positive patients through heterosexual transmission provided by China CDC to retrospect the transmission dynamics of HIV virus over time, and we estimated the origin time of four subtypes. The results show that the four subtype sequences calculations runs converged after 200 million, 400 million, 300 million and 300 million iterations respectively. We neglected the first 10% of output as the burn-in. The effective sampling size (ESS) of all parameter estimations was usually several thousands and the minimum ESS was over 200.

**Fig 2:**
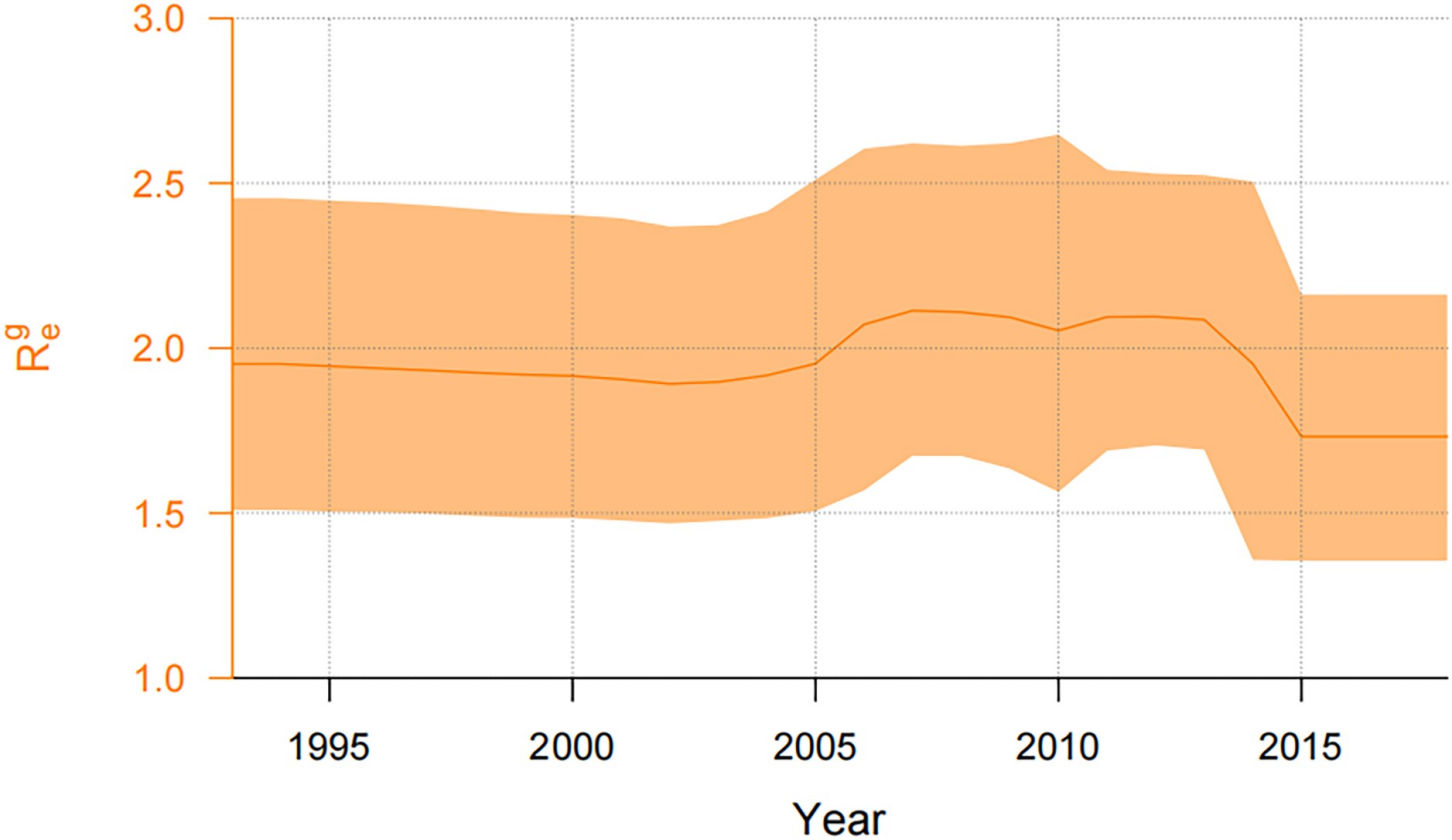
The weighted average of the effective reproductive number $R_{e}^{g}$ and its HPD interval.

Table 2 shows that the most recent common ancestor (tMRCA) of 350 CRF01AE subtype gene sequences is approximately 1994 (95% highest posterior density (HPD): 1991–1997). The tMRCA time of 322 CRF07BC subtype gene sequences is relatively earlier, around 1990 (95%HPD: 1986–1993). The tMRCA time of 206 CRF08BC subtype is about 1993 (95%HPD: 1991–1996). However, the tMRCA time of 34 CRFBC subtype is the earliest, about 1989 (95%HPD: 1979–1997). We also obtained the effective reproductive number $R_{e}$ over time from the gene sequences of four different subtypes (See Table 2). The *R*_*e*_ trends of all four subtypes are shown in the S1 Fig. We took the common part of the period for our research which is from 1994 to 2018. According to the weighted average of the corresponding $R_{e}$ values of the four gene sequences at the same time point, the value of the weighted average $R_{e}^{g}$ for heterosexual transmission of HIV/AIDS in China can be reached. Fig 2 shows the median line of $R_{e}^{g}$ and its 95%HPD interval from 1994 to 2018. Before 2005, the HIV/AIDS heterosexual transmission in China showed a relatively stable trend since the value of $R_{e}^{g}$ remained stable and slightly decreased, with a median around 1.90 (95%HPD: 1.38 -2.44). During the period from 2005 to 2014, $R_{e}^{g}$ rose steadily, then fell and finally rose again. During this period, it fluctuated slightly around 2.09 (95%HPD: 1.64–2.62). After 2014, $R_{e}^{g}$ remained stable, where the value is maintained at approximately 1.73 (95%HPD: 1.36–2.16).

**Table 2:** Estimation of each subtype and its 95%HPD interval.

Subtypes	Parameter estimation	tMRCA	Effective reproductive number $R_{e}^{i}$					
			$R_{e}^{1}$	$R_{e}^{2}$	$R_{e}^{3}$	$R_{e}^{4}$	$R_{e}^{5}$	$R_{e}^{6}$
CRF01AE	95%HPD Lower	1991	1.70	1.66	1.67	1.78	2.21	1.37
	Median	1994	2.09	2.04	2.06	2.15	2.59	1.63
	95%HPD Upper	1997	2.50	2.44	2.48	2.54	2.98	1.90
CRF07BC	95%HPD Lower	1986	1.57	1.52	1.55	1.60	1.56	1.47
	Median	1990	1.94	1.87	1.90	1.95	1.90	1.81
	95%HPD Upper	1993	2.31	2.23	2.26	2.31	2.25	2.16
CRF08BC	95%HPD Lower	1991	1.18	1.17	1.05	1.67	1.18	1.18
	Median	1993	1.86	1.85	1.70	2.39	1.69	1.81
	95%HPD Upper	1996	2.60	2.58	2.40	3.20	2.22	2.51
CRFBC	95%HPD Lower	1979	1.23	1.21	1.24	1.27	1.16	1.24
	Median	1989	1.92	1.91	1.90	1.96	1.78	1.96
	95%HPD Upper	1997	2.69	2.67	2.62	2.71	2.47	2.75

### HIV/AIDS epidemic trend prediction

It’s unrealistic to avoid parameter estimation when performing prediction with dynamics models. Values of some conventional parameters can be derived from the empirical literature. Some conventional parameters in the four nested models are being treated as constant and the value details and references can be found in Table 3. Some may vary a lot due to different countries or different treatment therapies, so the parameter values cannot be simply quoted.

**Table 3:** Constant parameters.

Symbols	Parameters	Value	Source
*μ*	natural mortality rate	1/60	[16]
λ_1_	Proportional coefficient of infection rate of single-drug resistant strain carriers	0.685	[37]
λ_2_	Proportional coefficient of infection rate of dual-drug resistant strain carriers	0.59	[37]
λ_3_	Proportional coefficient of infection rate of triple-drug resistant strain carriers	0.15	[37]
1/*θ*_1_	The duration of the sensitive strain carriers in the first stage of infection	4	[16]
1/*θ*_2_	The duration of the sensitive strain carriers in the second stage of infection	6	[16]
1/*θ*_3_	The duration of the sensitive strain carriers in the third stage of infection	2	[16]
*ϵ*_1_	The probability of transmission was reduced after the first infection stage treatment	0.66	[16, 38]
*ϵ*_2_	The probability of transmission was reduced after the second infection stage treatment	0.66	[16, 38]
*ϵ*_3_	The probability of transmission was reduced after the third infection stage treatment	0.12	[16, 38]

The target population of this study is the heterosexual population in China. We obtained the estimated value of the effective reproductive number $R_{e}^{g}$ from 1994 to 2018 from gene sequence data. According to the “Next-generation operator” method in the literature [39], we can calculate the basic reproduction number $R_{0}^{d}$ for each sub-model of the corresponding historical period. Let $R_{e}^{d}=R_{0}^{d}*S\left(t\right)/N\left(t\right)$ [40], then correspondingly we got the effective reproduction numbers $R_{e}^{d}$ for each dynamic sub-model I, II, III, or IV. Therefore some uncertain parameters can be fitted between the two effective reproductive numbers ($R_{e}^{g}$ and $R_{e}^{d}$) of *the same historical period* by the Markov Chain Monte Carlo (MCMC) method. After running 5 million iterations, each parameter converges and the fitting results are shown in the Table 4. The third column of Table 4 shows the mean values of parameters and their 95% confidence intervals (95%CI). The last column (I, II, III, IV) shows the historical period from which the parameter was fitted in Table 4. The figures of two *R*_*e*_ fitting in the corresponding historical period are shown in the S2 Fig. The parameters in Tables 3 and 4 will be used as “baseline parameters” for subsequent predictions.

**Table 4:** Fitted parameters by MCMC for dynamics model.

Symbols	Parameters	Mean (95%CI)	Historical period
*a* ^*w*^	Female input rate	10509000 (6759900,14258100)	I
*a* ^*m*^	Male input rate	11011000 (6968300,15053700)	I
*c* ^*w*^	Average number of female partners per year	2.2765 (2.0120,2.5410)	IV
*c* ^*m*^	Average number of male partners per year	2.2799 (2.0142,2.5457)	IV
*δ*	withdrawal rate in each stage	0.1267 (0.0835,0.1699)	II
*β*_1_	Infection rate of sensitive strains carriers in the first stage of infection	0.1665 (0.1412,0.1919)	I
*β*_2_	Infection rate of sensitive strains carriers in the second stage of infection	0.0760 (0.0581,0.0939)	I
*β*_3_	Infection rate of sensitive strains carriers in the third stage of infection	0.1425 (0.1021,0.1828)	I
*k*	Infection rate ratio coefficient of female with respect to male	0.6504 (0.5650,0.7350)	I
*ρ*_1_	Proportion of patients receiving treatment in stage 1 infection	0.4484 (0.1960,0.7007)	IV
*ρ*_2_	Proportion of patients receiving treatment in stage 2 infection	0.5395 (0.2897,0.8750)	III
*ρ*_3_	Proportion of patients receiving treatment in stage 3 infection	0.7029 (0.6003,0.9257)	II
1/*ω*_1_	The course of the sensitive strain carriers after first stage of treatment	7.8624 (6.3078,9.417)	IV
1/*ω*_2_	The course of the sensitive strain carriers after second stage of treatment	11.358 (10.0691,12.6469)	III
1/*ω*_3_	The course of the sensitive strain carriers after third stage of treatment	4.0636 (3.4934,4.6338)	II
*γ*_1_	Proportion of single resistance discontinuation after treatment at each stage	0.0489 (0.0199,0.0779)	II
*γ*_2_	Proportion of dual resistance discontinuation after treatment at each stage	0.0503 (0.0212,0.0794)	II
*γ*_3_	Proportion of triple resistance discontinuation after treatment at each stage	0.0051 (0.0022,0.0079)	II

To implement the “Healthy China 2030” project and strengthen the medical and health system reformation, China’s “13th Five-Year Plan for Combating and Prevention of AIDS” in 2017 set the “90–90-90” goal. That is, the proportion of infected individuals detected by testing should be above 90%; the proportion of diagnosed infected individuals receiving antiviral treatment should be above 90%; the treatment success rate of infected individuals receiving antiviral treatment should be above 90% [41]. Under the current condition, the diagnosis rate is 68%, while 32% of those infected have not been identified. The treatment rate in the first, second and third infection stage is 44.8%, 54.0% and 70.3%, respectively. The infection rate after the first and second stages of treatment is 0.66 times less than that before treatment, and the infection rate after the third stage treatment is 0.12 times than that before treatment, which is still far from the “90–90-90” target. Since the first 90% (the diagnosis rate) is hard to implement, we consider the other two factors that are easy to control: 90% treatment rate and 90% treatment success rate. Thus our “ideal” state means that the two 90% are reached at the same time.

To compare the prevention and control effects of different measures, we will discuss the following three situations respectively: **S**_**1**_: Under baseline parameters (Tables 3 and 4); **S**_**2**_: Only increase the treatment rate to 90% (the target treatment rate); and **S**_**3**_: In an “ideal” state, the treatment rate reaches 90% and the treatment success rate reaches 90%. See Table 5 for specific parameters.

**Table 5:** Predicting assumptions.

Infectious stage	Scenario S_1_	Scenario S_2_	Scenario S_3_
The first infectious stage	*ρ*_1_ = 0.448, *ε*_1_ = 0.66	*ρ*_1_ = 0.9, *ε*_1_ = 0.66	*ρ*_1_ = 0.9, *ε*_1_ = 0.1
The second infectious stage	*ρ*_2_ = 0.540, *ε*_2_ = 0.66	*ρ*_2_ = 0.9, *ε*_2_ = 0.66	*ρ*_2_ = 0.9, *ε*_2_ = 0.1
The third infectious stage	*ρ*_3_ = 0.703, *ε*_3_ = 0.12	*ρ*_3_ = 0.9, *ε*_3_ = 0.12	*ρ*_3_ = 0.9, *ε*_3_ = 0.1

We predicted the transmission dynamics of drug resistance through heterosexual transmission in the next few years in China and studied feasible measures to prevent and control the spread of HIV resistance based on the prediction results. We simulated and predicted several important indicators reflecting the spread of drug resistance during 2019–2025: the number of new HIV infections, the number of drug-resistant HIV infections, the proportion of primary resistance to new infections, and the total drug-resistant rate, etc. In each case, 5000 samples are taken for important parameters. The mean value as well as 95%CI of the studied indicators are calculated according to the sampling results. Besides, the results are visualized.

#### Prediction of HIV/AIDS epidemic trend in China under baseline parameters

**Fig 3:**
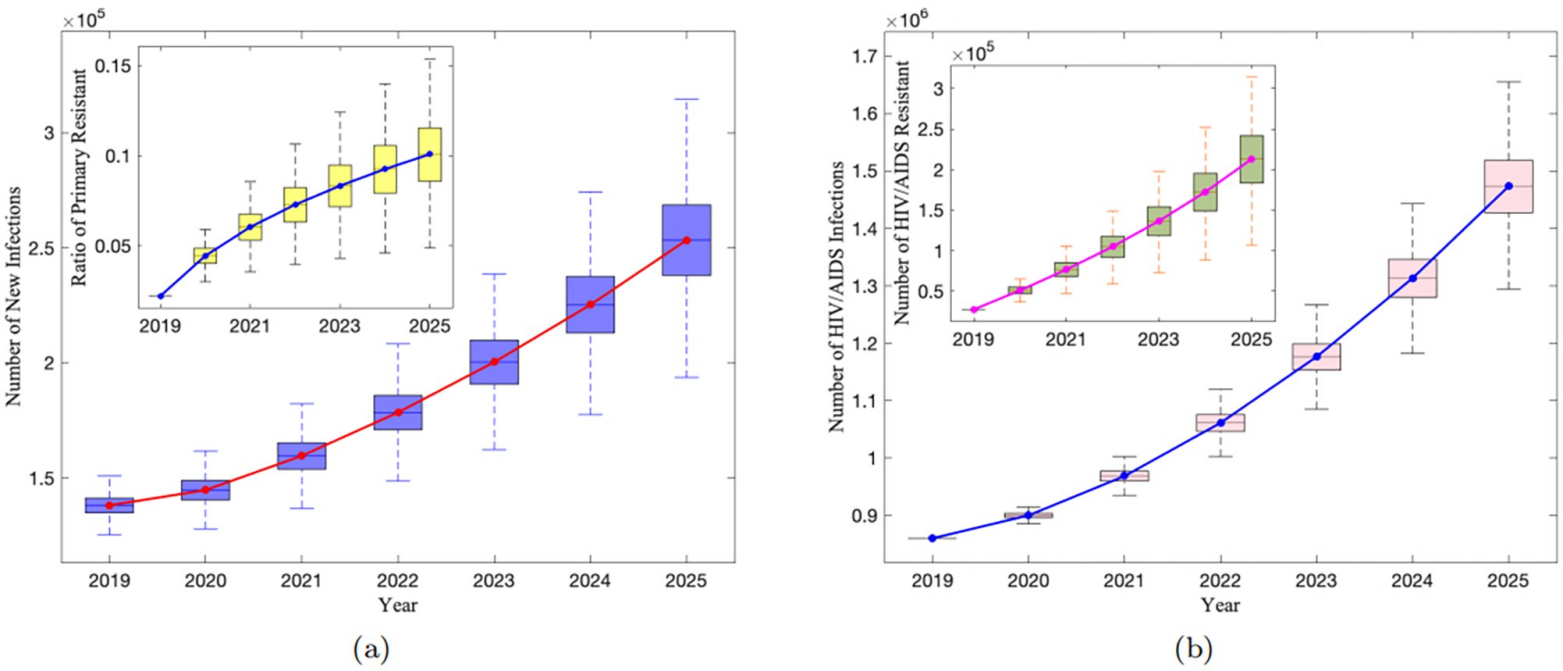
HIV/AIDS infection and drug resistance of heterosexuals under baseline parameters in China. a: The new infections and primary resistant rate. b: The total of HIV/AIDS infections and the total of HIV resistance.

Firstly, we studied the dynamic process over time in the heterosexual population using baseline parameters (i.e., no intervention measures). The results are shown in Fig 3.

Fig 3 shows that the four indicators of the epidemic are showing an upward trend. By 2025, the total number of new infections will reach 253422 (95%CI: 238000–268700, Fig 3(a) red line). The proportion of primary resistance in new infections will also rise to 10.1% (95%CI: 8.59%-11.56%, Fig 3(a) blue line). This means that one out of every 10 newly infected individuals is carrying drug-resistant strains. The total surviving infections increased from 860294 in 2019 to 1473478 in 2025 (95%CI: 1427000–1518000, Fig 3(b) blue line). The number of resistance among the infections has increased from 27530 in 2019 to 213250 in 2025 (95%CI: 183700–242000, Fig 3(b) purple line), which means the number of resistance in China will increase by nearly 8 times in few years. In the other words, the resistance rate will be as high as 14.48% (95%CI: 12.42%-16.50%) by 2025. The above data shows one clear fact: the HIV/AIDS epidemic and resistance among heterosexuals in China have not been effectively controlled.

#### Influence of intervention measures on HIV/AIDS epidemic trend

**Fig 4:**
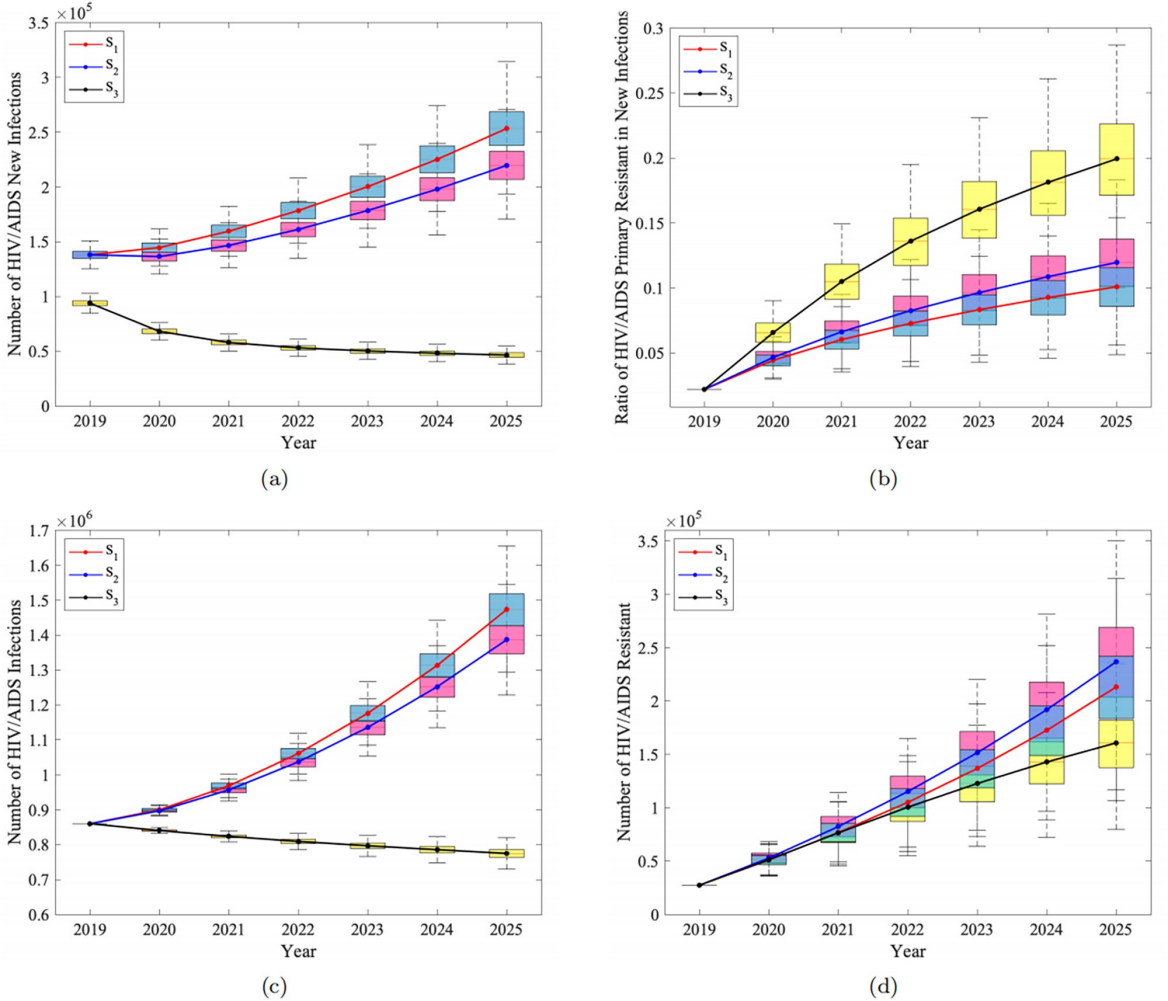
Prediction of HIV prevalence and drug resistance under different intervention measures. a: The total new infections. b: The rate of primary resistance in new infections. c: The total of infections. d: The number of HIV resistance.

Among HIV/AIDS intervention measures, we considered increasing the treatment rate to the target treatment rate (i.e. case **S**_**2**_) for diagnosed patients in 2019. The results are shown in Fig 4.

It can be seen from the Fig 4 that total new infections and the proportion of primary drug resistance in new infections under **S**_**2**_ is still showing an upward trend (Fig 4(a) and 4(b) blue curve). By 2025, the number of new infections will reach 219,717 (95%CI: 206900–232600), which is only slightly lower than that of **S**_**1**_. The proportion of primary resistance in new infections is 11.97% (95%CI: 10.14%-13.77%), which is higher than that of **S**_**1**_. In addition, we also predicted the total infections in 2025 under **S**_**2**_ (Fig 4(c) blue curve) and the number of resistance infections (Fig 4(d) blue curve). They both show an upward trend by 2025 and the total infections will reach 1387031 (95%CI: 0.1176%-0.1246%), which is only slightly lower than that under **S**_**1**_. The increase of resistance rate under **S**_**2**_ can be as high as 17.09% (95%CI: 14.60%-19.50%), which is faster than that under **S**_**1**_. The prediction results indicate that only increasing the treatment rate can only reduce the epidemic slightly, but will lead to more serious resistance problems.

Since merely increasing the treatment rate has little effect on controlling the epidemic, we hypothesized if the new drugs can achieve the desired therapeutic effect in addition to the increased treatment rate (i.e.**S**_**3**_). The results are shown in the black curve in Fig 4a–4d. As we expected, the trend of new infections can change significantly, from the original upward trend to a clear downward trend. By 2025, the number of new infections will drop to 46,559 (95%CI: 44510–48710). Unfortunately, the resistance situation is just the opposite. The proportion of primary resistance in new infections will greatly rise to 19.94% (95%CI: 17.13%-22.61%) in 2025. A similar trend can be found in the total resistance rate, which will rise to 20.76% (95%CI: 18.03%-23.17%).

To further investigate the drug resistance status, we compared the changes in the number of infected individuals and the number of drug resistance under three different intervention strategies, and the results are shown in Fig 4(c) and 4(d). We found that the trend of the number of infected individuals under three different strategies (Fig 4(c)) is similar to the number of new infections (Fig 4(a)). In 2025, compared with **S**_**1**_ and **S**_**2**_, the number of infected individuals under the strategy **S**_**3**_ can be significantly reduced to 775139 (95%CI: 763700–786300), which shows a great control effect. However, the curve for a number of drug resistance individuals (Fig 4(d)) and the curve for resistance rate are essentially different under the three different strategies. The number of resistance under ideal treatment effect (**S**_**3**_ in Fig 4(d), black curve) is actually reduced compared to baseline parameters (**S**_**1**_, red curve) and only increased the treatment rate (**S**_**2**_, bule curve). By 2025, the total number of resistance cases under the strategy **S**_**3**_ will be reduced to 160,899 (95%CI: 137700–182200), and the number of primary resistance compared with **S**_**1**_ and **S**_**2**_ will also be reduced to 9282 (95%CI: 7624.563–11013). Compared with the baseline parameters and only increasing the treatment rate, the drug-resistant rate increases but the number of resistance infections decreases under the ideal treatment effect, indicating that the proportion of drug-resistant strains among infected individuals becomes greater under this condition. Although the overall HIV prevalence has decreased at this time, drug-sensitive infections have decreased even more, leading to an increase in the resistance rate, but the number of resistance is actually a decline in a macroscopic view. Therefore, a comprehensive analysis of the two indicators of ratio and quantity can enable us to derive the impact of interventions: although ideal treatment effect should significantly reduce both HIV infection and the number of drug-resistant.

### Study on the multi-drug resistance dynamics under different intervention measures

**Fig 5:**
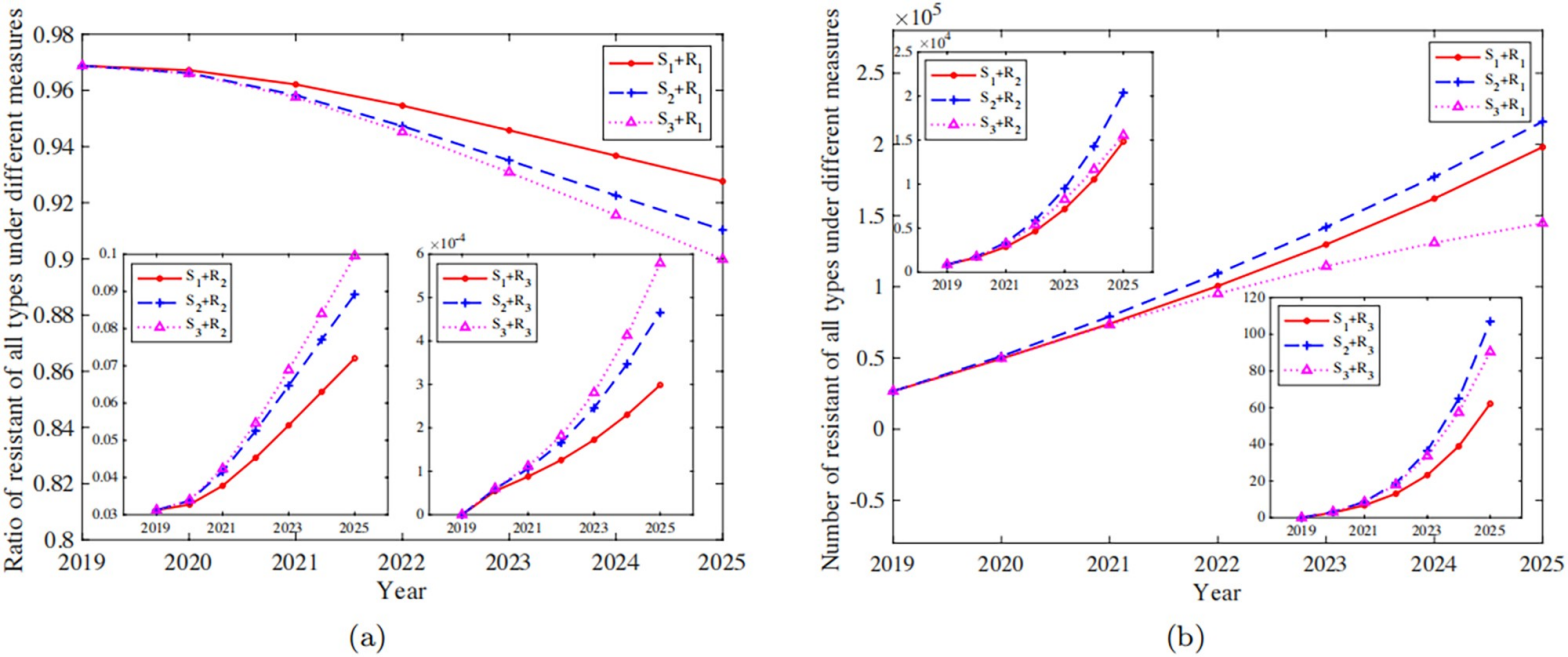
The multi-drug resistance dynamic under different intervention measures. a: The resistance rate of all types under different measures. b: The resistance number of all of all types under different measures.

The ART for individuals infected with HIV/AIDS is regarded as “cocktail” therapy in China [42]. Different drug combinations may lead to the emergence of different drug-resistant strains under long-term treatment [43]. In the following study, we will subdivide the types of drug-resistant strains, and abbreviate single-drug resistance, dual-drug resistance and triple-drug resistance as **R**_**1**_, **R**_**1**_ and **R**_**3**_ respectively. The effects of different prevention and control measures (**S**_**1**_, **S**_**2**_, **S**_**3**_) on the resistance dynamics of the three types above are shown in Fig 5.

The results show that the proportion of single-resistant strains under the three different strategies is the largest, and there is a downward trend (Fig 5(a)). Under **S**_**1**_, as of 2025, the number of single-resistance infections reached 19,0700 (95%CI: 170100–225000), accounting for 92.76% (95%CI: 91.68%-93.89%) of the total number of drug-resistant infections. As a comparison, the number of dual-resistance infections reached 14,844 (95%CI: 12230–17660), accounting for 7.20% (95%CI: 6.08%-8.28%). The number of triple-resistance infections is relatively low as expected, only 62.16 (95%CI: 49.24–76.54). The proportion of single-drug resistance in **S**_**2**_ shows a slight downward trend, compared with the proportion of single-drug resistance in **S**_**1**_. However, the number of overall drug resistance and the proportion of multiple-drug resistance has increased, and it has also increased relative to **S**_**1**_. Compared with **S**_**1**_ and **S**_**2**_, the proportion of single-drug resistance and the number of single-drug resistance under **S**_**3**_ are both decreased(Fig 5 pink purple dotted line, **S**_**3**_ + **R**_**1**_). The number and proportion of double and triple resistance are slightly higher than that of **S**_**1**_, but is lower compared to **S**_**2**_. In other words, compared with the baseline parameters, these two interventions will trigger an increase in the proportion of multi-drug resistance (dual and triple).

The number of populations with resistant strains shows a gradually upward trend no matter what intervention measures are taken (Fig 5(b)). However, it is obvious that the number of dual and triple resistance individuals grows more rapidly. Only increasing the treatment rate (the blue dashed line in Fig 5(b)) compared to the baseline parameters (the red solid line in Fig 5(b)) will lead to an increase in the number of three types of drug resistance. On the other hand, the ideal treatment effect (the pink-purple dotted line in the Fig 5(b)) can significantly restrain this trend. Compared with the baseline parameters, the number of single-drug resistance under the ideal treatment effect is obviously reduced. Although the number of dual-drug resistance and triple-drug resistance is slightly higher than the baseline parameters, it is significantly lower than the predicted value under increasing the treatment rate separately.

At the same time, we also predicted the number of individuals with new drug resistance (the primary resistance) each year, and the results are shown in Table 6. It can be seen that the primary resistance infection is increasing year by year. Compared with the baseline parameters (**S**_**1**_+**R**_**1**_, **S**_**1**_+**R**_**2**_, **S**_**1**_+**R**_**3**_), only increasing the treatment rate will lead to the simultaneous increase of the three types of resistance each year (**S**_**2**_+**R**_**1**_, **S**_**2**_+**R**_**2**_, **S**_**2**_+**R**_**3**_). The ideal treatment effect **S**_**3**_ can greatly reduce the annual primary resistance infections. By 2025, the annual primary single-drug resistance will be reduced to 8379 cases (95%CI: 7153–9540, **S**_**3**_+**R**_**1**_), while primary dual resistance will be reduced to 873.7 cases (95%CI: 701.5–1058, **S**_**3**_+**R**_**2**_), and the primary triple resistance will be reduced to 1.428 cases per year (95%CI: 1.10–1.811, **S**_**3**_+**R**_**3**_).

**Table 6:** New drug resistance infection under different scenario.

Years	2020	2021	2022	2023	2024	2025
Scenario						
**S**_**1**_ + **R**_**1**_	6239.5	9297.9	12455.7	15882.3	19652.8	23837.5
(95%CI)	(5625,6871)	(8161,10480)	(10800,14150)	(13680,18130)	(16870,22530)	(20430,27460)
**S**_**1**_ + **R**_**2**_	179.5	308.9	495.5	750.9	1086.4	1510.2
(95%CI)	(161.9,198.8)	(268.5,350.6)	(420.0,572.9)	(627.6,880.5)	(899.8,1289)	(1241,1811)
**S**_**1**_ + **R**_**3**_	0.085	0.188	0.347	0.599	0.985	1.539
(95%CI)	(0.071,0.099)	(0.156,0.222)	(0.287,0.416)	(0.489,0.729)	(0.789,1.212)	(1.22,1.917)
**S**_**2**_ + **R**_**1**_	6185.3	9387.5	12704.4	16234.4	20039.6	24185.2
(95%CI)	(5520,6830)	(8139,10600)	(10870,14460)	(13810,18590)	(17010,23020)	(20470,27810)
**S**_**2**_ + **R**_**2**_	184.9	348.4	599.7	946.4	1401.0	1967.2
(95%CI)	(164.9,205.4)	(298.1,401.1)	(498.4,705.6)	(774.6,1132)	(1131,1689)	(1578,2386)
**S**_**2**_ + **R**_**3**_	0.0925	0.234	0.484	0.911	1.578	2.547
(95%CI)	(0.0768,0.108)	(0.192,0.276)	(0.391,0.586)	(0.722,1.123)	(1.233,1.975)	(1.974,3.222)
**S**_**3**_ + **R**_**1**_	4346	5852	6850	7539	8020	8379
(95%CI)	(3810,4879)	(5015,6681)	(5827,7837)	(6411,8613)	(6833,9152)	(7153,9540)
**S**_**3**_ + **R**_**2**_	132.5	234.8	370.3	527.8	698.7	873.7
(95%CI)	(115.9,149.9)	(198.7,273.1)	(306,438.3)	(430.6,634.2)	(564.6,843.4)	(701.5,1058)
**S**_**3**_ + **R**_**3**_	0.080	0.180	0.344	0.599	0.958	1.428
(95%CI)	(0.066,0.0927)	(0.147,0.213)	(0.276,0.416)	(0.471,0.740)	(0.745,1.204)	(1.10,1.811)

## Discussion

It is common knowledge that parameter estimation is the most critical step for the numerical simulation as well as the prediction of dynamic models. We have learned that virus sequences contain a large amount of information about the diseases transmission while previous studies often ignored the transmission information carried by genetic sequence data. In this study, we employed the genetic sequences sampled from infected individuals. We combined the virus evolution process with the population dynamics model, applied Bayesian phylogenetic analysis to estimate the parameters of the virus evolution and epidemiological history, and considered the actual treatment of free antiretroviral treatment in different historical periods of China. The method can be applied to individual programs or regional programs, and the dynamic model pattern is generally applicable, but the parameters need to be re-fitted according to the local sampling sequence.

China put forward the goal of three 90% in the “China’s 13th Five-Year Plan for Combating and Prevention of AIDS”. The challenge is very difficult for the first 90% [44]. Strategies to increase the diagnosis rate is urgently desired. We must further increase publicity on HIV/AIDS prevention as well as its harm to the general public. In terms of the second and third 90%, they may be feasible. Nevertheless, our research found that if the treatment rate reaches 90% only, the infection rate and the number of new infections will still show an increasing trend. Also with the increase of total resistance rate and primary resistance rate, the number of primary and secondary resistance are increasing too. That is, as the number of treatments increases, serious drug resistance problems will occur. If the last two 90% are both achieved at the same time, the infection will be significantly reduced and show a downward trend. Although the resistance rate will increase, the number of resistance will be significantly reduced.

Furthermore, drug resistance is also subdivided in our model. China’s first-line drug treatment mainly involves three types of antiretroviral drugs. Due to the use of different drug combinations for targeted treatment, different types of drug resistance may occur. Drug resistance is a serious threat to the treatment of infections [45]. And Multi-drug resistance has caused great limitations in the choice of subsequent treatment options, so we want to control the spread of resistance while focusing on controlling the generation of multi-drug resistance. As the treatment rate increases, the number of single-drug resistance, double-drug resistance, and triple-drug resistance cases will all increase, and the number of multiple (double and triple) resistances will increase much faster than that of single resistance. If only the treatment rate is increased, the follow-up will lead to prevalent drug-resistance problems. The ideal treatment effect can greatly improve the resistance problem caused by only increasing the treatment rate. Under this measure, the number of single-drug resistance decreased, and the increase of the number of double-drug resistance and triple-drug resistance slowed down. In response to the HIV/AIDS epidemic in China, increasing only the treatment rate will not only fail to effectively control the epidemic situation, but also bring more complex drug resistance problems. Therefore, it is necessary to consider combining other measures to prevent and control HIV/AIDS. Besides, we need to monitor to the changes in multiple indicators to assess the effectiveness of the measures.

The study had the following limitations. First, HIV transmission among men who have sex with men (MSM) is gradually rising in China in recent years. Given that MSM are forced by public opinion to marry and have children with women in China, the spread of the virus between the two populations is intertwined [46–48]. If the MSM population is included, the model will be more complicated than the current one. Second, in terms of resistance classification, three major types of resistance (NRTI, NNRTI, PI) in China’s first-line ART were considered in the model, but the addition of new antiretroviral drugs (such as integrase inhibitors, InSTI) was not considered. Although InSTI is not yet used in first-line therapies, it may be provided in the future. Therefore, we still need to consider the effect of the addition of other drug on the model. The addition of InSTI will significantly improve the therapeutic effect of existing drugs and more effectively reduce the viral load in infected, thus reducing the new infections $\Lambda_{j}^{l}\;\left(j=0,1,2,3\right)$ in the model. The combination of InSTI with other antiretroviral drugs may solve the problem of resistance to a single drug and cross-resistance, thus reducing the resistance rate *γ*_*i*_ (*i* = 1, 2, 3) in the model. Finally, data availability and recency are one of our limitations. In this current research, we used the previous official and validated data from China CDC. However, the China CDC has not released the most recent HIV/AIDS statistics that we can use to validate our prediction.

## Supporting information

S1 File Supplement data and results. The prior information setting for BDSKY and additional numerical simulation data.
(PDF)

S1 Fig The median of the effective reproductive number of four subtypes and its HPD interval. (TIF)

S2 FigThe two effective reproductive numbers ($R_{e}^{g}$ and $R_{e}^{d}$) fitting in corresponding historical periods.(TIF)
