# Supplementary material for: The transmission of drug-resistant strains of HIV in heterosexual populations based on genetic sequences
Source: PLoS One. 2021 Dec 1;16(12):e0259023. doi: 10.1371/journal.pone.0259023 (PMC8635345; doi:10.1371/journal.pone.0259023)
Supplement: S1 File — The prior information setting for BDSKY and additional numerical simulation data. (PDF) [file pone.0259023.s001.pdf]

1. Bayesian phylogenetics

Under the Bayesian inference framework, we used BDSKY to perform Bayesian phylogenetic analysis on the four subtype sequences. The important prior parameter distribution settings are as follows:

Table 1: The prior distribution of BDSKY

Subtypes	$\mathcal{R}_e^g$	Removal rate	Origin	Sampling time
CRF01AE	LogN(0.75,0.1)	LogN(-1.0,0.50)	LogN(3.20,0.25)	Beta(10,65)
CRF07BC	LogN(0.65,0.1)	LogN(-1.0,0.50)	LogN(3.20,0.25)	Beta(10,65)
CRF08BC	LogN(0.63,0.2)	LogN(-1.5,0.75)	LogN(3.30,0.20)	Beta(5,60)
CRFBC	LogN(0.65,0.2)	LogN(-1.2,1.00)	LogN(3.45,0.25)	Beta(5,60)

2. Initial Population

According to the 2018 National Population Statistics Yearbook and the National AIDS/STD Comprehensive Prevention and Control Data Information Annual Report, the initial population value of each compartment is calculated as shown in the following table 2.

Table 2: The initial population in 2019

Male compartment				Female compartment			
$S^m = 557326895$				$S^w = 572423231$			
$I_{10}^m = 55518$	$I_{11}^m = 1778$	$I_{12}^m = 57$	$I_{13}^m = 0$	$I_{10}^w = 55518$	$I_{11}^w = 1778$	$I_{12}^w = 57$	$I_{13}^w = 0$
$T_{10}^m = 83276$	$T_{11}^m = 2667$	$T_{12}^m = 86$	$T_{13}^m = 0$	$T_{10}^w = 83276$	$T_{11}^w = 2667$	$T_{12}^w = 86$	$T_{13}^w = 0$
$I_{20}^m = 55518$	$I_{21}^m = 1778$	$I_{22}^m = 57$	$I_{23}^m = 0$	$I_{20}^w = 55518$	$I_{21}^w = 1778$	$I_{22}^w = 57$	$I_{23}^w = 0$
$T_{20}^m = 83276$	$T_{21}^m = 2667$	$T_{22}^m = 86$	$T_{23}^m = 0$	$T_{20}^w = 83276$	$T_{21}^w = 2667$	$T_{22}^w = 86$	$T_{23}^w = 0$
$I_{30}^m = 55518$	$I_{31}^m = 1778$	$I_{32}^m = 57$	$I_{33}^m = 0$	$I_{30}^w = 55518$	$I_{31}^w = 1778$	$I_{32}^w = 57$	$I_{33}^w = 0$
$T_{30}^m = 83276$	$T_{31}^m = 2667$	$T_{32}^m = 86$	$T_{33}^m = 0$	$T_{30}^w = 83276$	$T_{31}^w = 2667$	$T_{32}^w = 86$	$T_{33}^w = 0$

3. Predicting data under other different measures

We also predict the infection and drug resistance data under other different measures from 2020 to 2025. These measures include increasing the treatment rate, reducing the number of sexual partners and increasing the efficiency of condom use. The specific measures are as follows:

(1)$\mathbf{M_1}$:The treatment rate is increased by 20%.

(2)$\mathbf{M_2}$:The effectiveness of condoms is increased by 20%.

(3)$\mathbf{M_3}$:The number of sexual partners is reduced by 20%.

The results are shown in Table 3, Table 4, Table 5 and Table 6. We have found that increasing the treatment rate can slightly reduce infections but will lead to an increase in the number of drug-resistant individuals, while reducing the number of sexual partners and increasing the efficiency of condom use can greatly reduce infections and reduce the number of drug-resistant individuals.

Table 3: The number of new infection under different measures

Measures \ Years	2020	2021	2022	2023	2024	2025
$\mathbf{S_1}$	144730	159636	178495	200359	225293	253422
(95%CI)	(140400,149000)	(153800,165300)	(171000,185900)	(190700,209900)	(213000,237600)	(238000268700)
$\mathbf{M_1}$	139485	151555	167869	186944	208593	232707
(95%CI)	(135100,143800)	(145900,157300)	(160500,175200)	(177700,196300)	(197000,220300)	(218300,247300)
$\mathbf{M_2}$	109161	113789	120590	128497	137119	146432
(95%CI)	(106000,112400)	(109900,117700)	(115800,125300)	(122700,134200)	(130300,143900)	(138500,154400)
$\mathbf{M_3}$	109199	113910	120713	128571	137259	146524
(95%CI)	(106100,112200)	(110000,117700)	(116100,125200)	(123000,134100)	(130600,143800)	(138800,154300)

Table 4: The ratio of primary resistance in new infection under different measures

Measures \ Years	2020	2021	2022	2023	2024	2025
$\mathbf{S_1}$	0.0444	0.0604	0.0729	0.08338	0.0927	0.1009
(95%CI)	(0.0402,0.0486)	(0.0531,0.0676)	(0.0632,0.0826)	(0.0717,0.0949)	(0.0793,0.1058)	(0.0859,0.1156)
$\mathbf{M_1}$	0.0457	0.0636	0.0781	0.0906	0.1016	0.1114
(95%CI)	(0.0412,0.0500)	(0.0558,0.0713)	(0.0676,0.0885)	(0.0778,0.1034)	(0.0867,0.1164)	(0.0947,0.128)
$\mathbf{M_2}$	0.0456	0.0634	0.0777	0.0900	0.1009	0.1108
(95%CI)	(0.0413,0.0499)	(0.0558,0.071)	(0.0675,0.0879)	(0.0775,0.1024)	(0.0865,0.1152)	(0.0945,0.1268)
$\mathbf{M_3}$	0.0456	0.0635	0.0778	0.0902	0.1011	0.1114
(95%CI)	(0.0413,0.0499)	(0.0558,0.071)	(0.0675,0.0880)	(0.0776,0.1025)	(0.0865,0.1154)	(0.0945,0.1271)

Table 5: The number of infection under different measures

Measures \ Years	2020	2021	2022	2023	2024	2025
$\mathbf{S_1}$	900211	968745	1061413	1176503	1313593	1473478
(95%CI)	(896500,903800)	(960100,977100)	(1046000,1076000)	(1153000,1199000)	(1280000,1346000)	(1427000,1518000)
$\mathbf{M_1}$	898448	961967	1046920	1151978	1276554	1421028
(95%CI)	(894700,902200)	(953300,970600)	(1032000,1062000)	(1129000,1175000)	(1244000,1309000)	(1376000,1465000)
$\mathbf{M_2}$	869072	898066	941232	995537	1059025	1130857
(95%CI)	(866200,871900)	(891700,904300)	(930600,951600)	(979900,1011000)	(1038000,1080000)	(1103000,1159000)
$\mathbf{M_3}$	869109	898184	941446	995880	1059570	1131301
(95%CI)	(866300,871900)	(892000,904200)	(931300,951300)	(980800,1010000)	(1039000,1080000)	(1104000,1158000)

Table 6: The number of resistance under different measures

Measures \ Years	2020	2021	2022	2023	2024	2025
$\mathbf{S_1}$	51187	76875	105213	136922	172694	213248
(95%CI)	(46920,55440)	(68260,85370)	(91940,118000)	(118700,154400)	(149200,195700)	(183700,242000)
$\mathbf{M_1}$	52251	80020	110707	144887	183414	226337
(95%CI)	(47750,56650)	(70870,89020)	(96460,124600)	(125300,163900)	(157800,207600)	(194200,256800)
$\mathbf{M_2}$	50118	73418	97451	122561	148840	176630
(95%CI)	(45850,54270)	(65030,81530)	(84990,109300)	(105900,138000)	(128100,168200)	(151500,200200)
$\mathbf{M_3}$	50102	73329	97303	122436	148774	176563
(95%CI)	(45890,54260)	(65110,81470)	(85070,109200)	(106100,137900)	(128400,168100)	(152300,200100)
