# Supplementary figures and images for: The transmission of drug-resistant strains of HIV in heterosexual populations based on genetic sequences
Source: PLoS One. 2021 Dec 1;16(12):e0259023. doi: 10.1371/journal.pone.0259023 (PMC8635345; doi:10.1371/journal.pone.0259023)

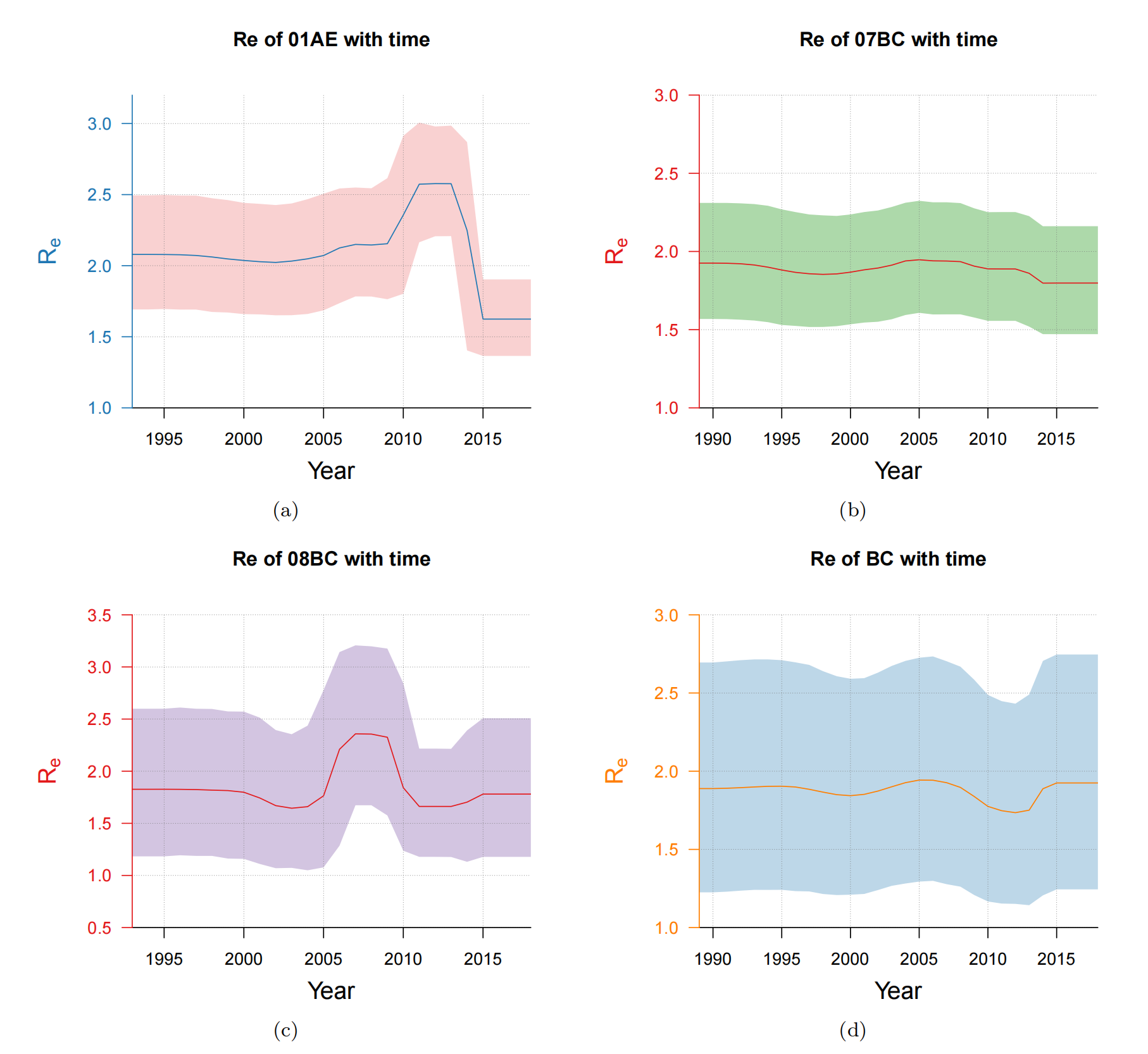

Supplement: S1 Fig — (TIF) [file pone.0259023.s002.tif]

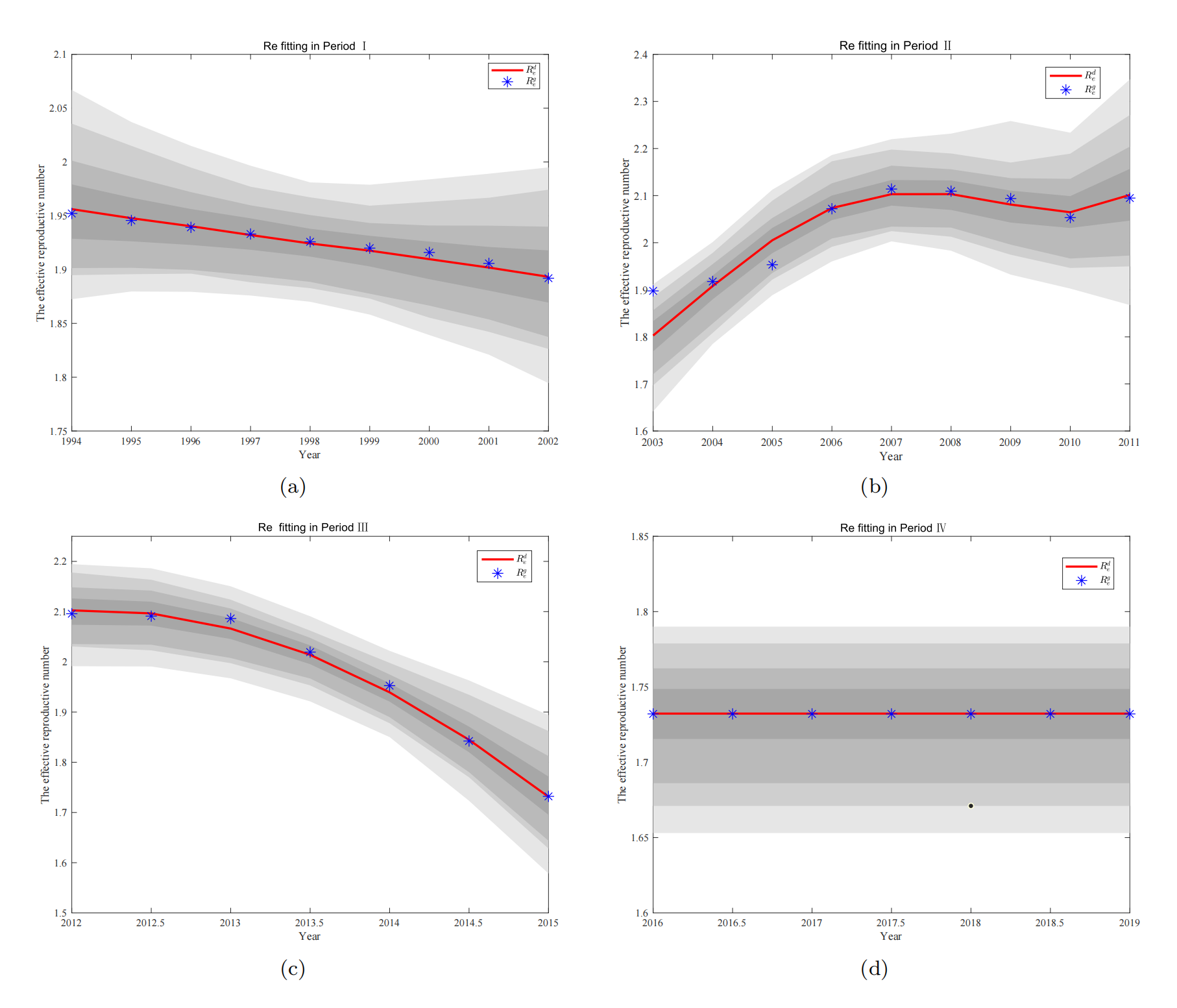

Supplement: S2 Fig — (TIF) [file pone.0259023.s003.tif]
